# Fabrication of ECM protein coated hollow collagen channels to study peripheral nerve regeneration

**DOI:** 10.1038/s41598-024-67046-1

**Published:** 2024-07-12

**Authors:** Jarin Tusnim, Karthik Budharaju, Jonathan M. Grasman

**Affiliations:** Department of Biomedical Engineering, New Jersey Institute of Technology, Newark, NJ 07102 USA

**Keywords:** Extracellular matrix proteins, Peripheral nerve regeneration, In vitro tissue models, Neuroengineering, Biomedical engineering, Biomaterials

## Abstract

Peripheral nerve injury is a prevalent clinical problem that often leads to lifelong disability and reduced quality of life. Although peripheral nerves can regenerate, recovery after severe injury is slow and incomplete. The current gold standard treatment, autologous nerve transplantation, has limitations including donor site morbidity and poor functional outcomes, highlighting the need for improved repair strategies. We developed a reproducible in vitro hollow channel collagen gel construct to investigate peripheral nerve regeneration (PNR) by exploring the influence of key extracellular matrix (ECM) proteins on axonal growth and regeneration. Channels were coated with ECM proteins: collagen IV, laminin, or fibronectin and seeded with dorsal root ganglia (DRG) collected from E16 rat embryos to compare the ability of the ECM proteins to enhance axonal growth. Robust axonal extension and Schwann cell (SC) infiltration were observed in fibronectin-coated channels, suggesting its superiority over other ECM proteins. Differential effects of ECM proteins on axons and SCs indicated direct growth stimulation beyond SC-mediated guidance. In vitro laceration injury modeling further confirmed fibronectin’s superior pro-regenerative effects, showcasing its potential in enhancing axonal regrowth post-injury. Advancing in vitro modeling that closely replicates native microenvironments will accelerate progress in overcoming the limitations of current nerve repair approaches.

## Introduction

The nervous system is a crucial component of the body and damage to this system can result in serious or potentially lethal consequences. In particular, the peripheral nervous system (PNS) is susceptible to injury because of the extensive presence of nerves throughout the body. Nerve injuries can have major consequences on patient quality of life because they can result in sensory and/or motor function deficits^1^. Despite breakthroughs in surgical technology, peripheral nerve injury (PNI) remains highly pervasive in modern society with twenty million recorded cases of patients in the US per year resulting from injuries such as motor vehicle accidents, toxins, and autoimmune diseases^2^. The gold standard for peripheral nerve repair is autologous nerve transplantation^3–5^. While this technique is successful, it has some limitations such as donor site morbidity, the formation of an additional operation site, difficulty in matching recipient and host nerves, donor site denervation, and neuroma formation at the site of harvest^6–8^. Moreover, nerve transplantation yields relatively poor results: it is estimated that only 25% of patients restored full motor function and only 3% regained full sensory function after median nerve repair^9,10^. Therefore, there is a clear need to develop alternative strategies for the treatment of PNI to mitigate these disadvantages and improve patient quality of life.

Current research studying peripheral nerve development and regeneration are mostly conducted in vivo^11,12^*.* While the complexity of in vivo models can simulate human physiological conditions ^13^, this complexity can be a hindrance to the understanding of the role of individual components in a single biological event. Conversely, in vitro model systems offer enhanced control and monitoring, facilitating detailed analysis of cellular responses and reducing the need for animal experimentation. Thus, developing in vitro model systems for peripheral nerve regeneration (PNR) is of significant interest for screening new treatments and enhancing our understanding of neuronal network formation and regeneration^14^. While there are some two-dimensional (2D) in vitro models present to study PNR, they cannot capture the three-dimensional (3D) architecture and spatial organization found in tissues and organs which can affect cell behavior, differentiation, and response to stimuli, highlighting the necessity for 3D models in PNR research^15^. Available 3D models, such as those using neuro-spheroids^15^ or collagen-chitosan sponges^16^, have shown promise but still face limitations such as lack of linear guidance cues and optical opacity. A notable model system in this space is the human nerve-on-a-chip model by AxoSim^17^, however, it lacks the incorporation of extracellular matrix (ECM) proteins essential for nerve development and regeneration. Our approach involves creating hollow channels within 3D hydrogels to better mimic native nerve structures, allowing for the evaluation and comparison of ECM proteins in a physiologically relevant environment^18^. Using type I collagen (“collagen”) hydrogels, which replicates the native extracellular space and structural properties of nerves^19,20^, our model provides optical transparency for non-destructive imaging^18^. Therefore, our model provides an opportunity to expand fundamental understanding of how axons initially infiltrate and progressively innervate along collagen channels, incorporating native ECM elements.

The ECM microenvironment is crucial for providing structural support, signal transduction, and also plays a significant role in the growth and guidance of regenerating nerves^21^. Key ECM proteins in peripheral nerves include laminin, fibronectin, and collagen IV^22–25^, which influence Schwann cell (SC) proliferation, migration, and myelination^26–29^. SCs provide trophic support to axons via expression of various growth factors and hormones, especially after nerve injury^30,31^. In addition to signaling SCs, growth cones from injured axons directly anchor themselves to ECM proteins to extend and ultimately reconnect with their efferent targets^28^. Therefore, it is important to investigate the influence of ECM proteins in both neuronal development and regeneration. Despite extensive research on the roles of ECM proteins in neuronal development and regeneration, there are conflicting reports on their relative effectiveness. For example, fibronectin-enriched conduits improved muscle reinnervation compared to laminin^32^, while laminin-coated hydrogels enhanced nerve regeneration and remyelination more than fibronectin^23,33^. Another study found no significant differences between laminin and fibronectin on axonal outgrowth and SC proliferation^26^, highlighting the need for a direct side-by-side analysis of ECM components on substrates that mimic the collagen-rich axonal niche in the PNS to better understand their contributions within a more relevant biomimetic model for studying PNR.

Therefore, we propose to leverage our 3D model system to address these critical gaps to understand the relative impact of each ECM protein on axonal growth and regeneration inside physiologically relevant microenvironments. We fabricated hollow channels within collagen hydrogels, where the channel mimics the physical space through which regenerating axons would navigate^18^. These studies aim to strategically incorporate collagen IV, laminin, and fibronectin into the channels to study the effects of these proteins on axonal growth and SC migration. Finally, we evaluated the ability of this system to serve as an in vitro injury model to study the role of ECM proteins on axonal regrowth. The goal of the research was to create a suitable in vitro 3D tissue system to model peripheral nerve development and compare the ability of ECM proteins to enhance axonal growth and regrowth after injury to study PNR.

## Methods

### 2D collagen hydrogel fabrication and coating

Type I rat tail collagen (Corning, Corning, NY) was combined with 10X phosphate buffered saline (PBS; Fisher Scientific, Hampton, NH), growth medium (DMEM/F-12 (Gibco, Gaithersburg, MD) medium supplemented with 10% FBS (Gibco) and 1% anti-anti (Gibco)), and sodium hydroxide (NaOH; Sigma-Aldrich, St. Louis, MO) on ice per the manufacturer’s instructions to form collagen hydrogels at a final concentration of 6.5 mg/mL. To optimize the concentration of protein coatings prior to subsequent 3D studies, 200 µL of collagen hydrogels were cast on the bottom of 24 well plates and polymerized for 1 h at 37 °C followed by incubation with either 0, 1, 10, or 100 µg/mL collagen IV (Corning); 0, 1, 10, or 50 µg/mL laminin (Corning); or 0, 5, 50, or 200 µg/mL fibronectin (Corning) mixed with DMEM/F-12 (Gibco) for 1 h at 37 °C to coat the 2D gels. After coating, the ECM protein solution was aspirated, and the gels were rinsed 2 times with growth medium at 37 °C.

### Compression testing of collagen hydrogels

To determine the stiffness of the bulk collagen hydrogel, we performed unconfined compression testing using an Instron machine (Instron Corp., Norwood, MA) where pre-formed collagen hydrogels (final concentration: 6.5 mg/mL, 8 mm in diameter and 6 mm in height) were placed in a petri dish containing 1X PBS. Hydrogels were tested using a parallel plate geometry at room temperature, where the gels were placed under a preload of 0.5 N to ensure full contact between the hydrogel and loading surface using a 10 N load cell. Samples were compressed to 50% strain at a rate of 0.6 mm/min. Force and displacement values were continuously recorded until the end of the test. Stress was calculated by dividing force by the initial cross-sectional area of the hydrogel and strain was calculated as the displacement from the initial height of the hydrogel (6 mm). Stress vs. strain graphs were plotted, and the tangent modulus was calculated between 10 and 20% strain via a custom MATLAB script.

### Fabrication of hollow channel collagen gels for 3D study

**Figure 1:**
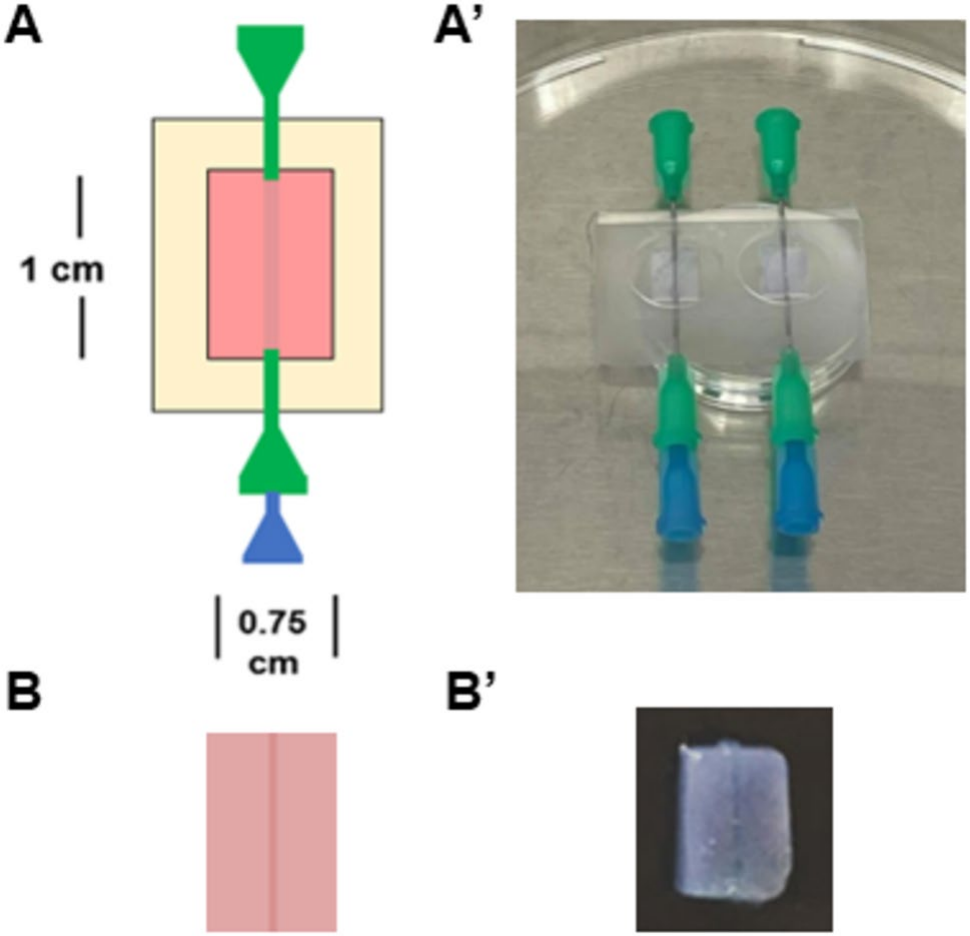
Overview of fabrication of 3D collagen gel with hollow channel. (**A**) 21G Blunt-end needles (green in color) were inserted into a polydimethylsiloxane (PDMS) mold and a 25G blunt end needle (blue in color) was threaded through the pair of 21G needles. A collagen gel was cast around the needles. (**B**) The 25G needle was removed after polymerization to create hollow channels. Channels were coated in various ECM proteins, where needed. They were incubated and the gel was removed from the PDMS mold into 24 well plates for culture. In all cases, panels with prime designations are representative photographs of their respective schematics.

Hollow channel collagen gels were fabricated as previously described^18^. Briefly, a custom polycarbonate mold was created by machining 0.9 cm tall cuboidal posts 1 cm × 0.75 cm. The outer walls were machined with a 45° angle to facilitate ease of removing constructs from the mold. Polydimethylsiloxane (PDMS; Sylgard 184; Dow Corning, Midland, MI) was mixed at a 10:1 (*w/w*) ratio of base to curing agent, degassed for at least 1 h, poured into the polycarbonate mold, and degassed for an additional 30 min. After curing at 60 °C for at least 8 h, the PDMS was peeled from the mold along the inclined walls, which made a total of two wells. After sterilization via autoclave, one pair of sterile 21G blunt-end needles (McMaster-Carr, Robbinsville, NJ) were affixed on either side of the 1 cm × 0.75 cm wells in aseptic conditions. A 25G blunt end needle (McMaster-Carr) which has an outer diameter of ~ 500 µm was threaded through each pair of 21G needles. To generate the bulk of the 3D tissue constructs, collagen (Corning) was mixed to a final concentration of 6.5 mg/mL as described above. The mixed collagen solution was pipetted into PDMS wells to cover the 25G needles with approximately 400 µL of solution, resulting in a final gel height of 2 mm, and incubated at 37 °C for 1 h to induce gelation (Fig. 1A,A’). After gelation, the 25G needles were carefully removed, resulting in the formation of hollow channels. To coat channels, ECM proteins (collagen IV, fibronectin, or laminin) at their optimized concentrations determined in 2D studies were suspended in serum-free DMEM/F-12 and injected into the hollow channels using a 1 mL syringe (Becton Dickinson Medical, Franklin Lake, NJ) and were incubated at 37 °C for at least 1 h. After coating, the hydrogel constructs were detached from the PDMS frames (Fig. 1B,B’) and transferred into 24 well plates filled with cell culture medium.

### DRG isolation and culture

All animal protocols were approved by the Institutional Animal Care and Use Committee (IACUC) at NJIT and Rutgers-Newark, and all procedures and methods were performed in accordance with relevant institutional guidelines and regulations and in accordance with the Animal Research: Reporting of In Vivo Experiments (ARRIVE) guidelines^34^. DRGs were isolated from E16 rat embryos immediately after American Veterinary Medical Association (AVMA) approved euthanasia, as previously described^35^. Briefly, under aseptic conditions, tissue from the embryo was dissected away to expose the spinal cord. DRGs were removed from the lumbar region of each embryo using fine-pointed forceps and transferred to a petri dish containing DMEM/F-12 supplemented with 1% anti-anti. DRGs were pooled from each litter, trimmed to remove any surrounding fascia, and subsequently cut in half using microscalpel blades and randomly selected to be seeded on top of the gels using pipettes for 2D studies or injected inside hollow channels for 3D studies using a 25G needle affixed to a syringe and cultured in growth medium for 5 days.

For 2D studies, no more than three DRG explants were seeded on top of the collagen gels in each well. For 3D studies, 4–5 DRGs were injected into hollow channels using a 25G needle affixed to a 1 mL syringe. For the laceration model, 5–6 DRGs were injected into the central region of the channels and cultured for 5 days prior to injury to allow them to grow and form axons. For each study, 2–3 independent litters were used to enhance scientific rigor. After the indicated culture times, samples were fixed with 4% paraformaldehyde (Boston BioProducts, Ashland, MA) and rinsed with PBS before processing for immunostaining.

### Development of injury model

Laceration-based injuries were conducted as we previously described^18^. In brief, DRGs were first seeded inside hollow channels and cultured for 5 days to allow for neuronal network growth. Subsequently, the hydrogels were cut with surgical scissors to create an in vitro injury, taking care to cut between DRG explant bodies without actually cutting the explants or dislodging them from the channels. Another collagen gel with hollow channels was fabricated using the same PDMS mold, cut in the same location as the DRG-seeded constructs, and fused with the wounded construct using a small amount of 3.5 mg/mL collagen gel. Care was taken to ensure that the two channels were properly lined up and that the coating condition of both DRG seeded and the newly attached constructs were the same. Fused constructs were cultured in growth medium and incubated at 37 °C for 1 or 2 weeks, after which they were fixed with 4% paraformaldehyde for 90 min to prepare for immunostaining to characterize axonal ingrowth into the new channel after injury.

### Immunostaining

For 2D studies, after fixation with 4% paraformaldehyde and subsequent rinses with PBS, tissue constructs were permeabilized with 0.1% (*v/v*) Triton X-100 (Sigma) in PBS for 20 min and rinsed with PBS supplemented with 0.05% (*v/v*) Tween-20 (Fisher Scientific) in PBS (PBST) 2 times for 5 min each. Samples were then blocked with 5% (*w/v*) bovine serum albumin (BSA, Sigma) in PBST for 30 min, and afterwards were immediately incubated with primary antibodies against β-tubulin III (1:500, Cat No. T2200, Sigma) in 1% (*w/v*) BSA/PBST for 1 h. Wells were washed several times with PBST and then incubated with species-matched Alexafluor 594 secondary antibodies (1:500, Cat No. A-11072, ThermoFisher) and 4',6-Diamidino-2-Phenylindole, Dilactate (DAPI; 1:1000, Cat No. D3571, Invitrogen) in 1% BSA/PBST for 30 min and finally rinsed with 1 × PBS three times for 5 min each before imaging.

For 3D constructs, after fixation with 4% paraformaldehyde and subsequent rinses with PBS, tissue constructs were permeabilized with 0.1% (*v/v*) Triton X-100 in PBS for 45 min and rinsed with PBST 3 times for 10 min each. Samples were then blocked with 5% (*w/v*) BSA/PBST for 1.5 h and afterwards were immediately incubated with a primary antibody against β-tubulin III (1:500) in 1% BSA/PBST, overnight, at room temperature. The following day, samples were washed several times with PBST, incubated with a species matched Alexafluor 594 secondary antibody (1:500), and DAPI (1:1000) in 1% BSA/PBST overnight, and the next day they were rinsed with 1X PBS five times before imaging.

To visualize SCs in our 3D constructs, we used the same protocol as above, except we used the following primary antibodies: β-tubulin III (1:500, Cat No. T8578; Sigma) and S100 polyclonal antibody (1:400, PA1-932, ThermoFisher), and appropriately species-matched secondary antibodies: Alexafluor 594 (1:300, A-11032A; ThermoFisher) and Alexafluor 488 (1:300, A-11070; ThermoFisher).

### Imaging and DRG axon length measurements

Fluorescent images were obtained using a Keyence BZ-X800 microscope (Keyence, Elmwood Park, NJ) and associated software using a 4X or 10X objective. Users were blinded to the samples they were imaging, as well as for subsequent analysis. To visualize DRGs or SCs seeded within the channels, the “full focus” feature of the Keyence microscope was utilized, which scanned through the thickness of the constructs and captured the pixels fully in focus for each image. The brightness and contrast of images were adjusted equally throughout the entire image and similarly across all images. Axonal lengths were measured using ImageJ software (NIH, Bethesda, MD). All the axons from each explant body were measured to obtain their lengths. Axons from explants that interacted with axons from other explants were excluded from analysis because of the uncertainty about whether the observed length belonged to a single axon or resulted from the contribution of axons from both explants. Next, we averaged the lengths from each explant to obtain average axonal lengths and identified the maximum axonal lengths from each explant body to obtain maximum axonal lengths. In all cases, the observer was blinded to the treatment group that was being imaged and analyzed. The size of each DRG body was also measured. While we typically did not observe any differences in the size of the DRGs, any DRG that did not have an easily defined DRG body, or a body that was smaller than 150 µm, was omitted from further analysis.

### Statistics

Data are presented as mean +/− standard deviation. Statistical analyses were performed using one-way analysis of variance (ANOVA) with Tukey post-hoc analysis using IBM SPSS Statistics (IBM, Chicago, IL). Where indicated, a Student’s t-test was performed using the same software. For all analyses, differences between experimental conditions were considered to be significant at p < 0.05.

## Results

### Optimization of ECM concentrations on 2D gels

**Figure 2:**
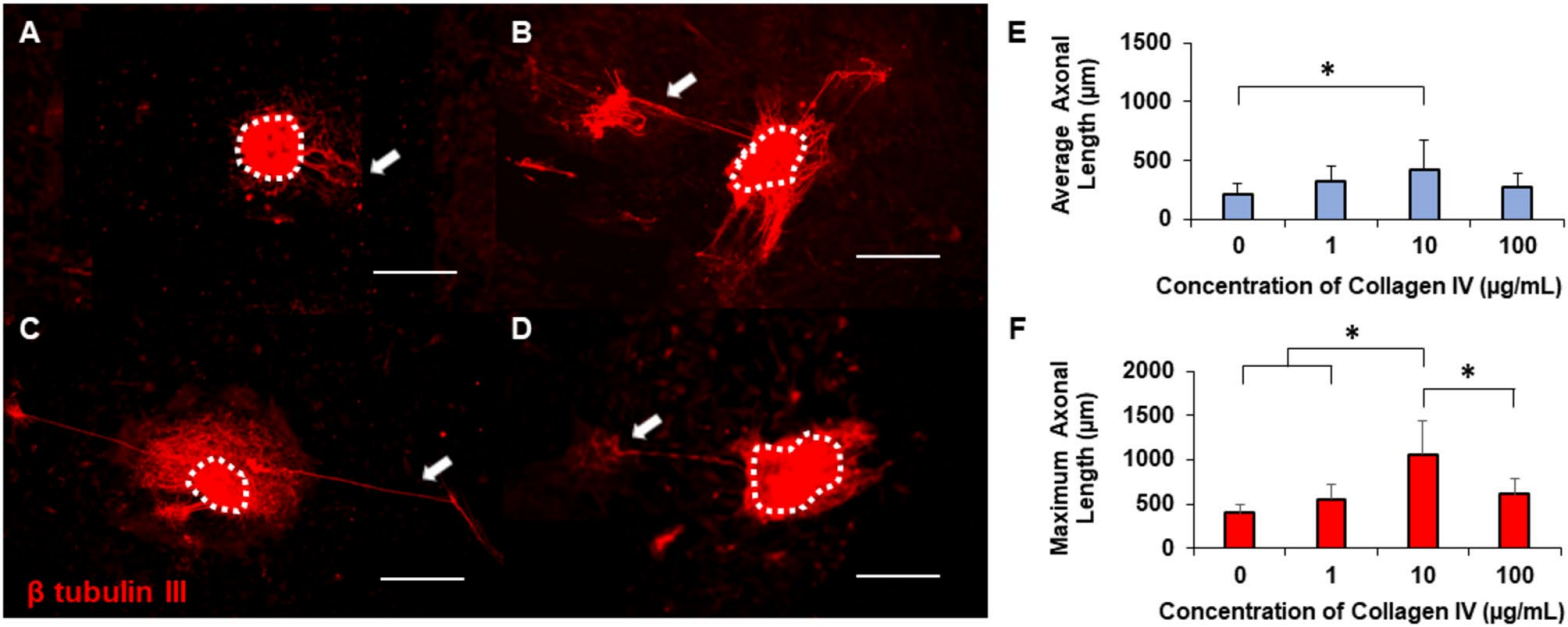
Representative images of axonal growth from DRGs on gels coated with (**A**) 0 µg/mL, (**B**) 1 µg/mL, (**C**) 10 µg/mL, or (**D**) 100 µg/mL collagen IV. Scale: 500 µm, dotted lines denote explant bodies, and arrows denote the longest axon extending from each DRG. Quantification of (**E**) average axonal length and (**F**) maximum axonal length on collagen IV coated gels. * indicates statistical significance between groups as determined by one-way ANOVA with post hoc Tukey test (p < 0.05); sample size, N > 12 for each group.

To quantitatively determine the effects of ECM protein concentration on axonal growth, increasing concentrations of specific proteins were first coated onto 2D collagen hydrogels. DRG explants isolated from E16 rats were grown on these substrates for 5 days, and the average and maximum axonal lengths were measured using ImageJ. Collagen hydrogels (6.5 mg/mL) were determined to have a compressive modulus of 2.5 ± 0.7 kPa, which is in the range of soft tissue and an optimal microenvironment to facilitate neuronal outgrowth^36,37^. Representative images using collagen IV coating demonstrate that long axons extend from individual explant bodies, particularly on coated surfaces (Fig. 2A–D). Generally, there were more axons visible on collagen IV coated surfaces than on control surfaces. However, the longest axons appeared to be single, or fasciculated, extensions from the explant, while numerous shorter axons were observed to extend around the explant bodies. Hydrogels coated with 10 µg/mL collagen IV showed longer axons extending from the explant body (Fig. 2C), in a similar, fasciculated manner. To obtain quantitative results on how the ECM coatings enhanced axonal growth, the average and maximum length of each axon from the explants were measured (Fig. 2-F). Collagen gels coated with 10 µg/mL collagen IV showed significantly higher average axonal growth than controls and qualitatively higher average axonal growth than the other experimental groups. There was a significant enhancement of maximum axonal growth on collagen gels coated with 10 µg/mL collagen IV with respect to all other treatment groups.

**Figure 3:**
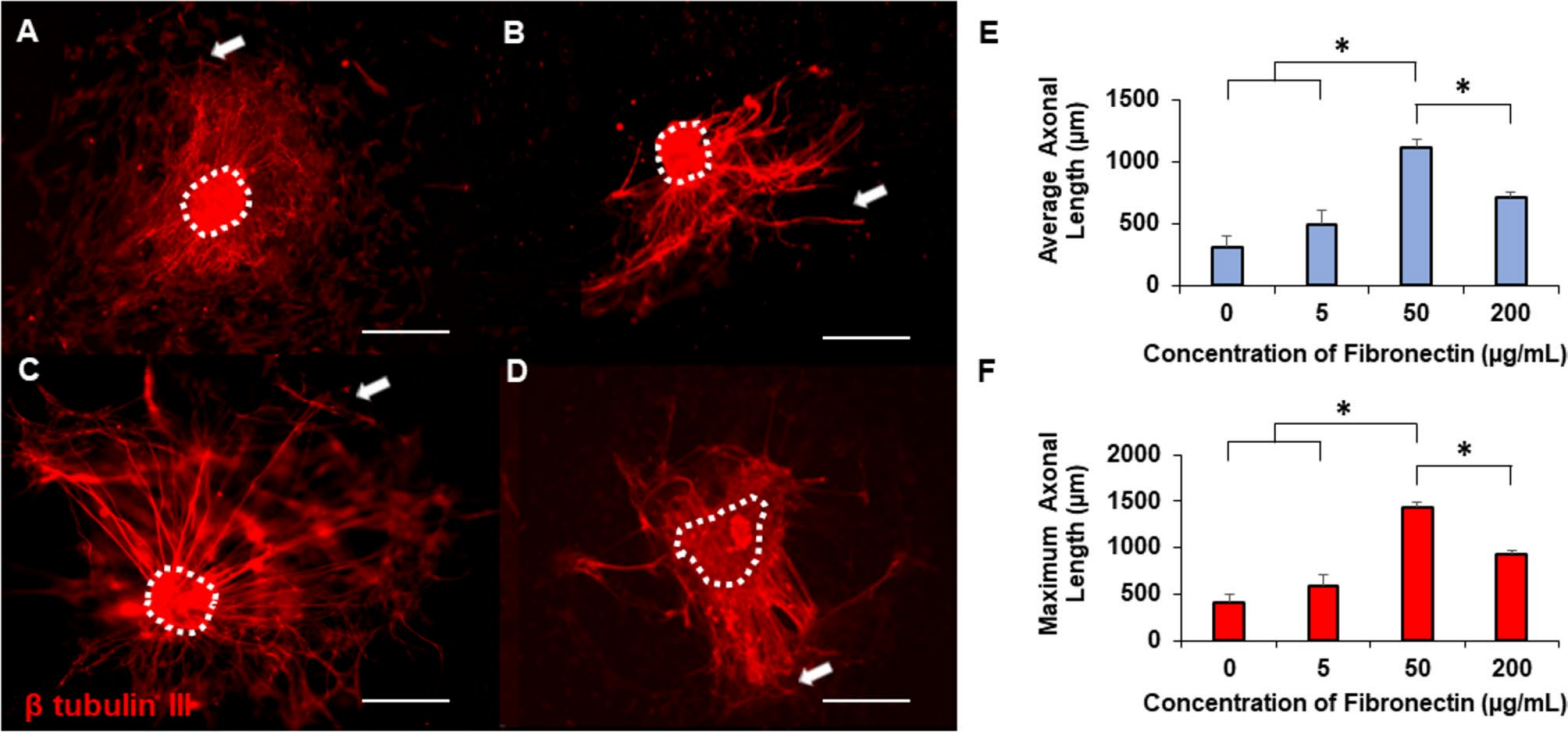
Representative images of axonal growth from DRGs on gels coated with (**A**) 0 µg/mL, (**B**) 5 µg/mL, (**C**) 50 µg/mL, or (**D**) 200 µg/mL fibronectin. Scale: 500 µm, dotted lines denote explant bodies, and arrows denote the longest axon extending from each DRG. Quantification of (**E**) average axonal length (**F**) maximum axonal length on fibronectin coated gels. * indicates statistical significance between groups as determined by one-way ANOVA with post hoc Tukey test (p < 0.05); sample size, N > 12 for each group.

Next, we investigated the effects of fibronectin on axonal growth. Representative images from fibronectin coated collagen gels showed improved axonal growth with fibronectin coating rather than uncoated collagen gels (Fig. 3A–D). Particularly, explants on collagen gels coated with 50 µg/mL fibronectin (Fig. 3C) exhibited longer axons and a higher number of axons compared to other conditions. Quantitative analysis of both average and maximum axonal lengths across varying fibronectin concentrations revealed that collagen gels coated with 50 µg/mL fibronectin resulted in significantly longer axonal growth with respect to all other treatment groups for both average and maximum axonal growth (Fig. 3E–F).

**Figure 4:**
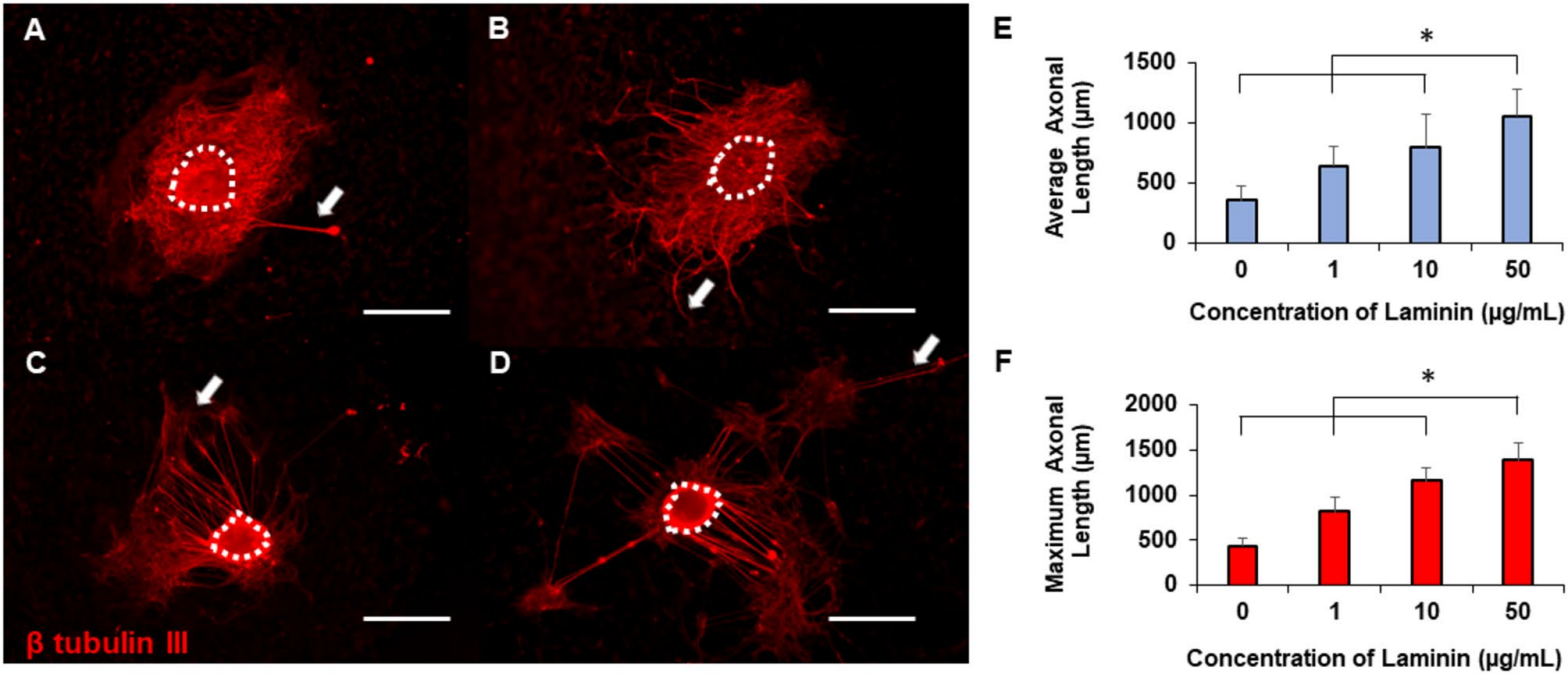
Representative images of axonal growth from DRGs on gels coated with (**A**) 0 µg/mL, (**B**) 1 µg/mL, (**C**) 10 µg/mL, or (**D**) 50 µg/mL laminin. Scale: 500 µm, dotted lines denote explant bodies, and arrows denote the longest axon extending from each DRG. Quantification of (**E**) average axonal length (**F**) maximum axonal length on laminin coated gels. * indicates statistical significance between groups as determined by one-way ANOVA with post hoc Tukey test (p < 0.05); sample size, N > 12 for each group.

Finally, we examined the effects of laminin coatings on axonal growth. Representative images from laminin coated collagen hydrogels reveal numerous axons extending from explant bodies seeded on increasingly higher concentrations of laminin (Fig. 4A–D). Particularly, 10 and 50 µg/mL of laminin supported not only numerous axonal extensions from the explants, but also longer axons as well. From the quantitative analysis (Fig. 4E–F) it can be concluded that 50 µg/mL laminin showed significantly higher average and maximum axonal length compared to all other concentrations. In this experiment, we identified the optimal concentrations of ECM proteins on collagen substrates that significantly enhance axonal growth. This emphasizes the importance of ECM concentration optimization before actual experimental evaluations.

### Fibronectin enhances axonal growth inside 3D hollow channels

**Figure 5:**
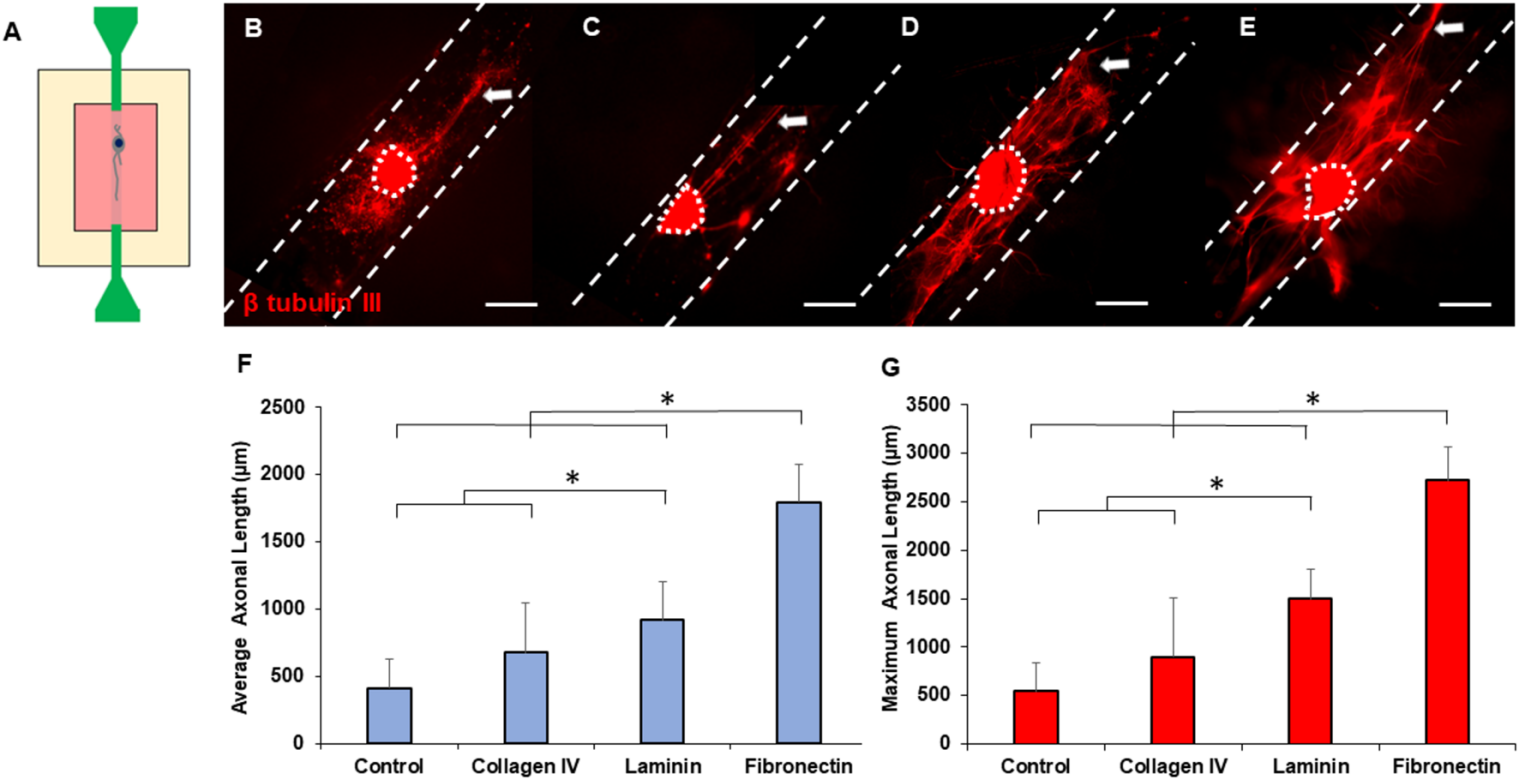
(**A**) Schematic of a DRG seeded hollow channel collagen gel construct. Representative images of axonal growth from DRGs inside the channels with (**B**) no-coating (control), (**C**) collagen IV, (**D**) laminin, and (**E**) fibronectin coating. Scale: 500 µm, dotted lines denote explant bodies, hatched lines show the channels, and arrows denote the longest axon extending from each DRG. Quantification of (**F**) average axonal length and (**G**) maximum axonal length for all coated and non-coated (control) 3D channels. * indicates statistical significance between groups as determined by one-way ANOVA with post hoc Tukey test (p < 0.05); sample size, N = 9 for each group.

To evaluate the potential of different ECM proteins to enhance axonal growth in a 3D environment, we seeded DRGs inside channels that were coated with the optimized concentrations of ECM proteins determined in our 2D study. Custom PDMS molds were used to fabricate hollow channels within collagen gels where DRG explant was seeded to facilitate axon formation (Fig. 5A), which have the same stiffness as our 2D hydrogels. Representative images from 3D studies show DRG explants attached to the inside of the channels with axons extending from the explant bodies (Fig. 5B–E). A modest number of relatively short axons were visible in control channels (Fig. 5B) and collagen IV coated channels (Fig. 5C), while qualitatively longer and higher numbers of axons were visible inside channels coated with laminin (Fig. 5D) and fibronectin (Fig. 5E), suggesting enhanced axonal growth. While laminin may have also supported axon defasciculation, the longest axons were observed inside fibronectin coated channels. We also observed some axonal growth into the bulk collagen gel material surrounding the channels, however, most of the axons appeared to prefer to grow within the hollow channels, suggesting that the hollow channels are providing contact guidance necessary to support axonal growth. To further substantiate our observations, we conducted quantitative assessments of average (Fig. 5F) and maximum (Fig. 5G) axonal lengths for each coating condition. For both metrics, laminin supported significantly longer average and maximum axonal lengths than control and collagen IV coatings, while fibronectin supported significantly longer axonal lengths than all other experimental conditions.

### ECM proteins stimulate Schwann cell outgrowth from explants

**Figure 6:**
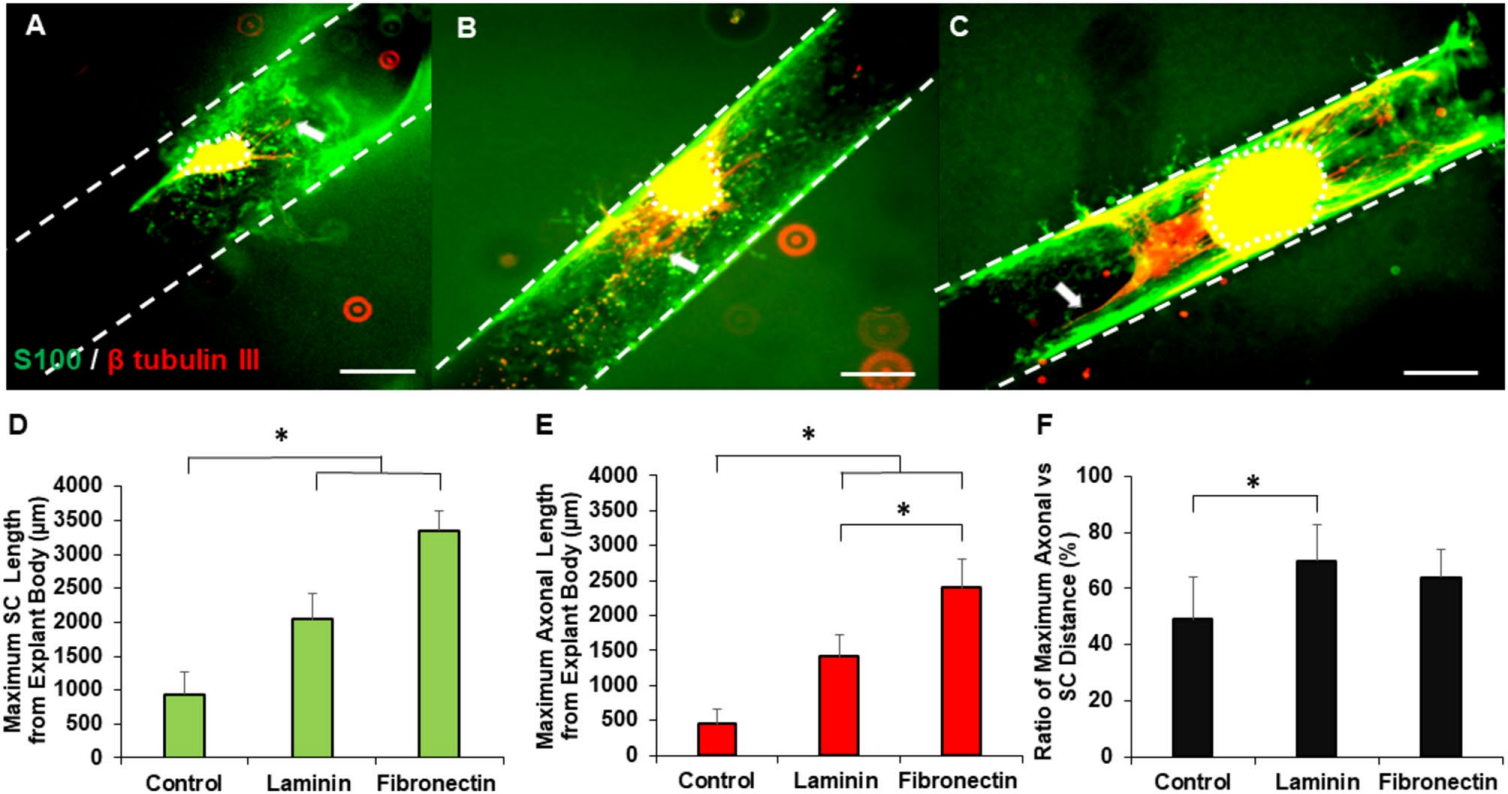
Representative images of Schwann cells and axons from explants in (**A**) control, (**B**) laminin, and (**C**) fibronectin coated channels after 5 days of culture. Scale: 500 µm, dotted lines denote explant bodies, hatched lines shows the channels, and arrows denote the longest axon extending from each DRG. Quantification of (**D**) maximum SC length, (**E**) maximum axonal length from DRG explants for all coated and non-coated channels, and (**F**) ratio of maximum axonal vs SC distance from explant bodies. * indicates statistical significance between groups by one-way ANOVA with post hoc Tukey test (p < 0.05); sample size, N = 9 for each group.

As SCs are important for peripheral nerve development and repair, we also analyzed the impact of ECM proteins on SC migration and outgrowth from DRG explants. We co-stained S100 (SC marker) with β tubulin III to observe colocalization between SCs and axons, as well as the independent growth of SCs with respect to the explants. In all cases, while SCs were observed to be in close proximity to sprouting axons, they also were distributed throughout the channel after 5 days in culture, suggesting that these cells migrated further than axons. Representative images reveal that SCs migrated longer distances from explants in the presence of fibronectin and laminin coatings with respect to controls (Fig. 6A–C). Qualitatively, fibronectin supported SC migration more than laminin as there was a higher density of SCs surrounding explants in fibronectin coated channels that also migrated longer distances from the explant bodies. These results are also consistent with those from our axonal outgrowth analysis where fibronectin coated channels supported higher axonal growth than laminin coated channels. To quantify these observations, we measured the maximum length that SCs traveled from the explant body (Fig. 6D), as well as the maximum axonal length (Fig. 6E). Both proteins significantly increased SC migration and axonal growth with respect to non-coated controls. Fibronectin coatings significantly enhanced axonal growth compared to laminin coatings, and qualitatively supported more SC migration than laminin coatings. To determine whether the effect of ECM proteins on axonal growth was solely due to their effect on SC migration, we measured the ratio of the maximum distance of axons from DRG explant to the maximum distance of SC migration from the DRG explant under each condition (Fig. 6F). Our findings revealed that this ratio is significantly higher in laminin coated channels and qualitatively higher in fibronectin coated channels compared to control.

### Fibronectin coated channel showed longer axonal growth post injury

**Figure 7:**
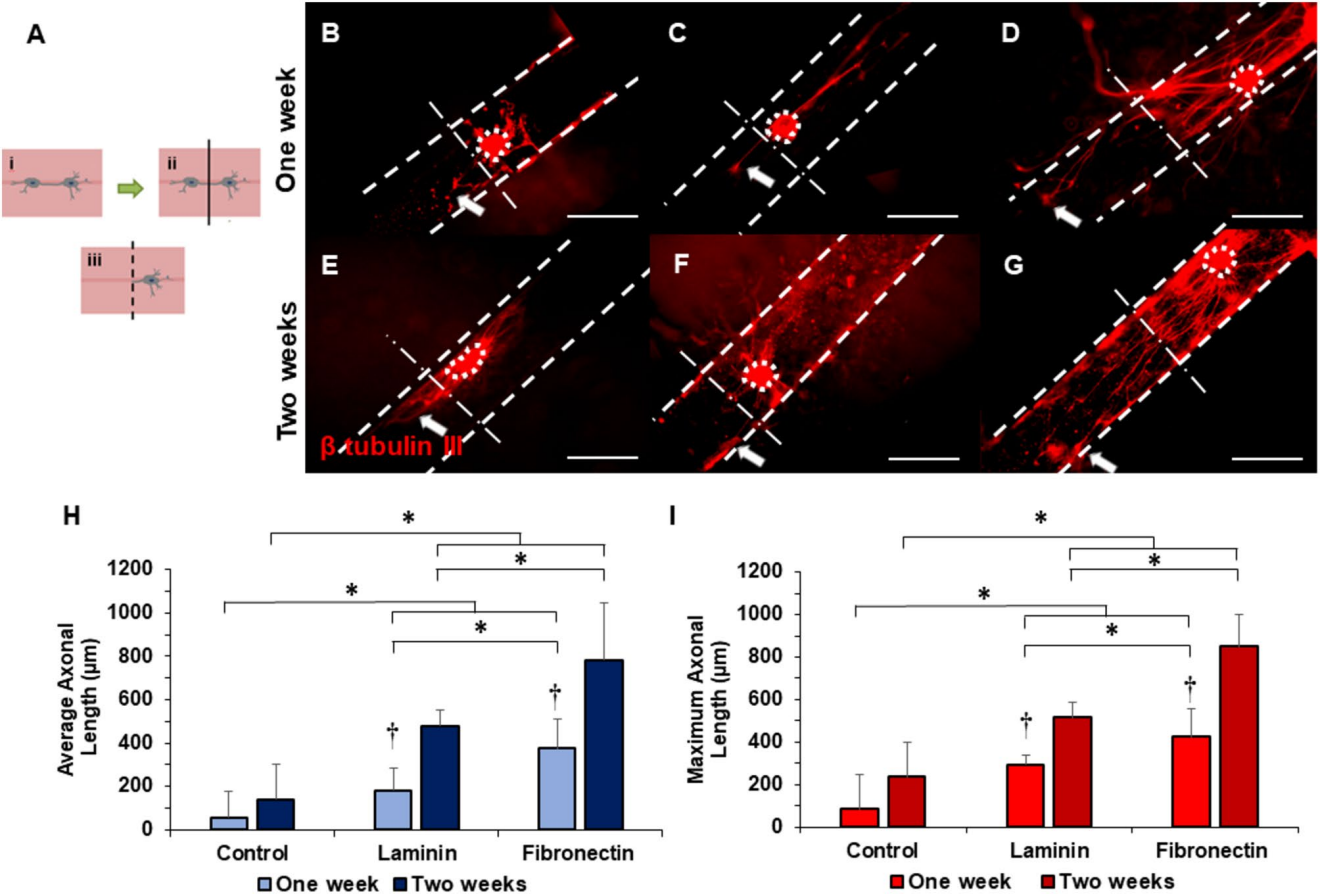
(**A**) Schematic of laceration model: (i) hollow collagen gel with seeded DRGs, (ii) cutting of axon after 5 days, and (iii) glued wounded construct and new acellular construct together. Representative images of axonal growth from DRGs after laceration: (**B**) non-coated (control), (**C**) laminin, and (**D**) fibronectin coating one week post injury, (**E**) control, (**F**) laminin, and (**G**) fibronectin coating two weeks post injury. Scale: 500 µm, dotted lines denote explant bodies, parallel hatched lines shows the channels, hatched-dotted lines represent the laceration plane, and arrows denote the longest axon extending into the new channel from each DRG. Quantification of (**H**) average axonal length and (**I**) maximum axonal length for all coated and non-coated (control) channels one week and two weeks post injury. * indicates statistical significance between groups as determined by one-way ANOVA with post hoc Tukey test (p < 0.05), and **†** indicates statistical significance between groups by Student’s t-test (p < 0.05); sample size, N > 6 for each group.

Finally, we generated a laceration injury model to determine the ability of ECM proteins to enhance axonal regeneration after a clinically relevant injury. Channels were seeded with DRGs and incubated for 5 days to facilitate axonal growth (Fig. 7Ai). After that, the entire gel, including the axonal network, was cut in half (Fig. 7Aii) and subsequently fused with an acellular construct to see whether axons would grow towards the new channel following injury (Fig. 7Aiii). Representative images from laceration studies reveal that axons grew into the new channels one week (Fig. 7B–D) and two weeks (Fig. 7E–G) post injury. In each image, channels are indicated with two parallel hatched lines, and the hatched-dotted line bisecting those parallel lines indicates the laceration plane; channels to the left of this line were initially acellular and channels to the right housed the injured explants. Therefore, all growth past this line indicates regrowth, or regeneration, at the indicated time points. The images reveal axonal growth into the new channels, indicating regrowth one and two weeks after injury. From the representative images, the control group exhibited minimal axonal regeneration into the new channel at either time point. Laminin coated channels improved regeneration compared to the control, supporting increased numbers of axons across the injury plane. Most notably, fibronectin-coated channels supported the most substantial axonal regrowth. Two weeks post-injury, numerous axons advanced beyond the injury line, indicating improved axonal regeneration compared to the other experimental conditions. We again observed the regenerating axons predominantly growing into the hollow channel of fibronectin coated channels, rather than progressing within the bulk collagen hydrogel. For the control and laminin coated channels, regenerating axons generally extended into the hollow channels as well. Quantitative analysis of the average (Fig. 7H) and maximum (Fig. 7I) axonal lengths of regenerating axons revealed significantly longer axons for both laminin and fibronectin treated channels with respect to controls at both time points. Fibronectin coated channels supported significantly longer axonal regrowth than laminin at both time points, suggesting that this protein is important both for initial axonal growth as well as for regrowth after injury. Axons grew significantly longer during two weeks of culture with respect to one week of culture after injury in channels coated with laminin and fibronectin. However, no such temporal significance was detected with control constructs. This suggests that the presence of ECM proteins, particularly fibronectin, may play a crucial role in promoting axonal regeneration post-injury. These findings provide valuable insights into the role of ECM proteins in axonal regeneration and may have significant implications for the development of therapeutic strategies for nerve injury repair.

## Discussion

In this study, our goal was to engineer 3D hollow channel constructs and determine the comparable impacts of ECM coating on axonal growth to ultimately enhance PNR. Using optimized concentrations of ECM proteins identified from 2D studies, we demonstrated that fibronectin enhanced axonal growth and SC migration, promoting more robust axonal regrowth in terms of average and maximum length after injury compared to the other ECM proteins investigated (laminin and collagen IV).

While laminin has conventionally been thought of as one of the most permissive ECM microenvironments for nerve development and regeneration, our results consistently showed the superior effects of fibronectin in axonal growth and regeneration. This observation contrasts with some existing literature, where laminin has been shown to be the most effective at enhancing neuronal outgrowth^33,38^. One reason for this divergence may stem from not optimizing ECM protein concentrations. Previous work comparing laminin with fibronectin used a fibronectin density of 10 µg/mL, which is lower than our optimized concentration^39^. From our 2D study, we found that the concentration of individual ECM proteins available to be coated onto the collagen substrate significantly impacted axonal growth and regeneration. Another reason could be the variable timeframes in which axonal growth was assessed. For instance, one study concluded that laminin coated scaffolds showed better axonal growth than fibronectin coated scaffolds after 1 day of culture. Interestingly, the same study also reported higher axonal growth rates on fibronectin-coated scaffolds than laminin coated scaffolds, which may suggest that fibronectin would ultimately support longer axons in subsequent days^33^. A final consideration for this divergence could be context-specific effects of the interactions between coating proteins and the bulk materials. For instance, Gonzalez-Perez et al. showed enhanced reinnervation and myelination with fibronectin compared to laminin on chitosan conduits^32^, while Siddique et al. found laminin to support longer axonal outgrowth than fibronectin on OPF hydrogels^33^, indicating that the impact of ECM proteins on nerve regeneration may vary in different microenvironments. Our results are consistent with the findings of Gonzalez-Perez et al.^32^. After injury, the growth cone of a regenerating axon binds to ECM proteins by integrins which facilitates axonal regeneration. The Rho proteins Cdc42, Rac1, and RhoA regulate the actin cytoskeleton^40,41^. Previous studies have demonstrated that integrin-mediated adhesion to fibronectin activates Cdc42 and Rac1, while inhibiting RhoA^40,42^. RhoA activation after nerve injury stiffens the cytoskeleton and eventually collapses the growth cone of axons, inhibiting regeneration^43^. Since RhoA suppression enhances axonal regeneration^44^, inhibition of this pathway by fibronectin may contribute to its regeneration of axons after PNI. Together, these data indicate that bulk-coating interactions must be taken into account to properly understand the impact of ECM proteins on axonal growth. Our approach was to load these proteins onto a collagen matrix with physiologically relevant stiffness values, revealing an important role for fibronectin in both axonal growth and regeneration following injury.

We consistently observed more axons growing inside the channel rather than into the bulk collagen gel. The ability of axons to selectively grow towards specific targets is crucial for successful nerve regeneration and suggests our model system shows promise for guiding axons for PNR applications. In the native physiological environment, axons rely on various guidance cues to navigate toward their appropriate targets during regeneration^45^. Mimicking these cues in tissue engineering approaches can enhance the regenerative capacity of injured nerves. Our channels appeared to replicate some of these directional cues. Due to the aligned tubular microarchitecture resembling the natural structure of nerves, axons may find it easier to populate this channel compared to the denser bulk region^46^. Additionally, ECM proteins are known to interact with cell adhesion receptors, such as integrins, and promote cell migration and axonal extension^47^. The presence of ECM proteins found in the native axonal niche within the hollow channels likely created a favorable microenvironment that attracted and guided axonal growth toward the channels. So, the incorporation of ECM proteins within the channels may play a significant role in guiding axonal growth.

SCs are crucial for peripheral nerve development and repair, and their migration plays an important role in guiding axonal growth. Our results demonstrated that SCs migrated further than axons in all conditions, indicating their potential to provide guidance cues for axonal growth. This observation is in line with previous work that demonstrated that SC signaling facilitates peripheral nerve repair by directing subsequent axonal growth^38^. Our results demonstrating that fibronectin coatings on collagen gels significantly enhanced SC migration aligns with a recent study that observed collagen enhancing SC viability, while fibronectin enhanced SC migration compared to laminin, poly-l-lysine, and poly-l-ornithine, and concluded that collagen and fibronectin are the most optimal surface materials for nerve guidance conduits^27^. Together, these findings suggest fibronectin has the ability to enhance SC motility, which may facilitate axonal growth by allowing glial cells to migrate ahead along the coated channels to stimulate axonal growth^27^. Interestingly, our findings also revealed that fibronectin, when coated on collagen, promotes axonal extension. The ratio of the maximum axonal growth and maximum distance of SC migration from the DRG explants varied between ECM proteins which suggests ECM proteins can separately impact both SC migration and axonal growth. Thus, even though SCs direct axonal growth, our data indicate that ECM proteins also have independent, direct, effect on promoting axonal extension. Neuronal integrins bind specific ECM ligands to regulate axon growth and pathfinding during development and regeneration^48^. For example, Melrose et al. mentioned that fibronectin and laminin directly regulate growth cone dynamics and neurite outgrowth through integrins^39^. This integrin-mediated binding inactivates RhoA and thus facilitates axonal outgrowth^40,42^. Studies also showed that ECM proteins can interact with growth factors and signaling pathways to promote or inhibit axonal growth^49^. Our findings that ECM coatings differentially affected axonal length and SC migration align with these studies that suggest ECM proteins have independent effects on axons beyond the guidance provided by SC migration.

While our model system aims to capture key aspects of the local microenvironment, there are some caveats. In particular, the immune system mediates inflammation and debris clearance after PNI^50^, but our model lacks the inclusion of immune cells such as macrophages. Moreover, unlike living models with circulation, our system does not replicate physiological blood flow. Beyond the inclusion of different cell types, we also do not deliberately texturize the walls of our channels, so there is an absence of topography that may be of use to further enhance axonal growth. Addressing these limitations may further enhance the ability of the model system to simulate the intricacies of the local microenvironment to study PNR. Recent advances in implantable nerve guidance conduits suggest that more sophisticated designs, such as multichannel conduits or hollow channels filled with antigliotic gels and growth factors, are more effective than autologous nerve transplantation and hollow nerve conduit in repairing long nerve gaps (> 2 cm) following PNI^51,52^. Thus, by incorporating fibronectin into existing practices, we can potentially enhance PNR outcomes in clinical settings, offering a readily translatable improvement to current therapeutic approaches.

## Conclusions

The significance of this study lies in uncovering the comparable contributions and effectiveness of ECM proteins in facilitating axonal growth and regeneration. We developed an in vitro 3D tissue system that serves as an effective model for simulating peripheral nerve development and axonal regeneration following injury. From our 2D experiments, we identified that 10 µg/mL collagen IV, 50 µg/mL laminin, and 50 µg/mL fibronectin supported the highest average axonal length and maximum axonal length when coated on top of a type I collagen matrix. We determined that fibronectin coated channels supported longer axonal growth and regeneration after injury than the other proteins when incorporated into 3D constructs designed to mimic the local microenvironment. Fibronectin also enhanced SC migration, suggesting its importance in all stages of neuronal growth. While laminin has conventionally been thought of as one of the most permissive ECM microenvironments for axonal growth and regeneration, our results showed an enhanced effect when coating fibronectin onto collagen constructs in peripheral nerve growth and regeneration. This could shift the paradigm in treatment strategies and open new avenues for research in nerve regeneration. Our findings provide valuable insights into the relative effectiveness of ECM proteins in PNR and may contribute to the development of novel therapeutic strategies for nerve injury repair. Future studies could focus on in vivo models to test the clinical viability of these findings, as well as investigate the potential synergistic effects of different ECM proteins or combinations with other growth factors in PNR.

## Data Availability

The authors declare that the data supporting the findings of this study are either available within the article or from the corresponding author upon request.
